# Outer Membrane Protein A Conservation among *Orientia tsutsugamushi* Isolates Suggests Its Potential as a Protective Antigen and Diagnostic Target

**DOI:** 10.3390/tropicalmed3020063

**Published:** 2018-06-11

**Authors:** Sean M. Evans, Haley E. Adcox, Lauren VieBrock, Ryan S. Green, Alison Luce-Fedrow, Suschsmita Chattopadhyay, Ju Jiang, Richard T. Marconi, Daniel Paris, Allen L. Richards, Jason A. Carlyon

**Affiliations:** 1 Department of Microbiology and Immunology, Virginia Commonwealth University Medical Center, School of Medicine, Richmond, VA 23298, USA; smevans@utah.gov (S.M.E.); adcoxhe@mymail.vcu.edu (H.E.A.); lviebrock@gmail.com (L.V.); greenrs@mymail.vcu.edu (R.S.G.); richard.marconi@vcuhealth.org (R.T.M.); 2 Viral and Rickettsial Diseases Department, Naval Medical Research Center, Silver Spring, MD 20910, USA; alfedrow@ship.edu (A.L.-F.); Suchismita.Chattopadhyay.ctr@mail.mil (S.C.); Ju.Jiang2.ctr@mail.mil (J.J.); Allen.L.Richards.civ@mail.mil (A.L.R.); 3 Department of Biology, Shippensburg University, Shippensburg, PA 17257, USA; 4 Department of Medicine, Swiss Tropical and Public Health Institute, 4051 Basel, Switzerland; daniel.paris@swisstph.ch; 5 Department of Preventive Medicine and Biostatistics, Uniformed Services University of the Health Sciences, Bethesda, MD 20814, USA

**Keywords:** scrub typhus, *Orientia tsutsugamushi*, *Rickettsia*, Rickettsiales, outer membrane protein A, *Anaplasma*

## Abstract

Scrub typhus threatens one billion people in the Asia-Pacific area and cases have emerged outside this region. It is caused by infection with any of the multitude of strains of the bacterium *Orientia tsutsugamushi*. A vaccine that affords heterologous protection and a commercially-available molecular diagnostic assay are lacking. Herein, we determined that the nucleotide and translated amino acid sequences of outer membrane protein A (OmpA) are highly conserved among 51 *O. tsutsugamushi* isolates. Molecular modeling revealed the predicted tertiary structure of *O. tsutsugamushi* OmpA to be very similar to that of the phylogenetically-related pathogen, *Anaplasma phagocytophilum*, including the location of a helix that contains residues functionally essential for *A. phagocytophilum* infection. PCR primers were developed that amplified *ompA* DNA from all *O. tsutsugamushi* strains, but not from negative control bacteria. Using these primers in quantitative PCR enabled sensitive detection and quantitation of *O. tsutsugamushi ompA* DNA from organs and blood of mice that had been experimentally infected with the Karp or Gilliam strains. The high degree of OmpA conservation among *O. tsutsugamushi* strains evidences its potential to serve as a molecular diagnostic target and justifies its consideration as a candidate for developing a broadly-protective scrub typhus vaccine.

## 1. Introduction

Scrub typhus is an acute, febrile, and potentially deadly disease caused by infection with the larval *Leptotrombidium* mite-vectored bacterium, *Orientia tsutsugamushi*. Long known to be endemic to the Asia-Pacific, a densely-populated region where more than one million cases are estimated to occur annually, scrub typhus affects all organs and the central nervous system. Clinical manifestations can include eschar, fever, myalgia, maculopapular rash, lymphadenopathy, pneumonitis, meningitis, and coagulopathies that can result in circulatory system collapse (reviewed in [1,2]). The disease can account for up to 20% of all acute undifferentiated febrile episodes and up to 27% of blood culture-negative fever patients in endemic areas [3,4,5]. Its non-specific clinical presentation makes clinical diagnosis very difficult [6]. Mortality rates range from less than 1.4% to approximately 70%, depending on prior patient immune status, whether proper antibiotic treatment is initiated in a timely manner, and the bacterial strain causing the infection [1,7,8,9]. Recent outbreaks in the Asia-Pacific [10,11,12,13,14,15,16,17,18,19,20,21,22,23] as well as evidence for scrub typhus or scrub typhus-like infections in Cameroon, Kenya, Congo, Djibouti, Tanzania, Chile, and Peru signify these illnesses as both emerging and reemerging diseases of global importance [24,25,26,27,28,29,30].

The genus *Orientia* is a member of the order Rickettsiales, which contains other arthropod vector-transmitted pathogens, including *Anaplasma*, *Ehrlichia*, and *Rickettsia*. Until recently, the genus consisted of a single species, *Orientia tsutsugamushi*, which contained multiple antigenically-distinct strains [31]. In 2010, a second agent, *Candidatus* Orientia chuto, was discovered in a febrile patient presenting with a scrub typhus-like illness that had been acquired in the United Arab Emirates [32]. The extensive immunogenic diversity among *O. tsutsugamushi* strains has contributed to the inability to develop a scrub typhus vaccine that achieves heterologous protection despite more than seven decades’ worth of efforts [6]. No commercially-available molecular diagnostic assay for the disease exists. Serology-based tests suffer from a high seroprevalence baseline among populations living in scrub typhus-endemic areas. While polymerase chain reaction (PCR)-based tests can overcome limitations of serologic assays, only a limited number of *Orientia* spp. nucleic acid sequences have been explored for their potential as molecular diagnostic targets [33,34,35,36,37].

Outer membrane protein A (OmpA; also referred to as peptidoglycan-associated lipoprotein) is conserved among most Gram-negative bacteria and contributes to the virulence of Gram-negative pathogens, especially their abilities to adhere to and invade host cells [38,39,40,41,42,43,44,45]. Antisera raised against entire OmpA proteins or specific binding domains thereof for *Anaplasma* spp., *Ehrlichia chaffeensis*, and *R. conorii* inhibit bacterial invasion of host cells in vitro [38,41,42,44,45]. These Rickettsiales members express OmpA during infection of human patients and/or experimentally infected animals [38,44,46]. Several Rickettsiales species and strains have stretches of *ompA* DNA sequences that exhibit high degrees of identity [44,45,47,48], which suggests their potential as effective nucleic acid-based diagnostic targets. Limited evidence suggests that OmpA antibodies offer at least some protection from rickettsial infections in vivo [49]. While *O. tsutsugamushi* Ikeda expresses OmpA during infection of mammalian host cells in vitro [50], *ompA* conservation among *Orientia* spp., and whether these bacteria express *ompA* during in vivo infection, have yet to be examined.

In this study, we determined that *ompA* DNA and translated amino acid sequences are highly conserved among 51 geographically-diverse *O. tsutsugamushi* isolates. Molecular modeling revealed the predicted tertiary structure of *O. tsutsugamushi* OmpA to be very similar to that of *Anaplasma phagocytophilum* OmpA, including the location of a helix and residues thereof that are essential for *Anaplasma* spp. OmpA function. A PCR primer pair was developed that amplified *ompA* DNA from all *O. tsutsugamushi* strains examined and enabled sensitive detection and quantitation of *O. tsutsugamushi ompA* DNA from organs and blood of experimentally-infected mice. The high degree of conservation of OmpA among *O. tsutsugamushi* isolates suggests that it be considered both as a diagnostic target and potential antigen for developing a broadly-protective scrub typhus vaccine.

## 2. Materials and Methods

### 2.1. O. tsutsugamushi DNA Samples Examined in This Study

Nearly all of the *O. tsutsugamushi* strains examined in this study have been previously described [32,51,52,53,54,55,56,57,58,59,60,61,62,63,64]. The isolates, their countries of origin, publication in which they were originally reported, and their *ompA* GenBank accession numbers and locus tags are listed in Table 1.

### 2.2. PCR, Cloning, and DNA Sequence Analyses

PCR was performed using isolated *O. tsutsugamushi* strain DNA and MyTaq polymerase Red (Bioline, Taunton, MA, USA) following the manufacturer’s instructions. Following an initial denaturing step at 95 °C for 1 min, thermal cycling conditions were 35 cycles of 95 °C for 15 s, 55 °C for 15 s, and 72 °C for 10 s, followed by a final extension at 72 °C for 20 s. Amplicons were analyzed in 2.0% agarose gels in 40 mM tris-acetate-2 mM EDTA (pH 8.5). Primer sequences targeting *ompA* were designed according to *ompA* (OTT_RS06375) of the annotated *O. tsutsugamushi* Ikeda genome [65] and are listed in Table 2. DNA samples that yielded amplicons of the expected sizes were again subjected to PCR using the appropriate primer sets and Platinum HiFi Taq polymerase (Thermo Fisher, Waltham, MA, USA) according to the manufacturer’s instructions. Platinum HiFi Taq polymerase thermal cycling conditions consisted of an initial denaturation step of 94 °C for 2 min, followed by 34 cycles of 94 °C for 15 s, 55 °C for 30 s, and a final extension step at 68 °C for 1 min. The resulting amplicons were subjected to agarose gel electrophoresis, after which they were visualized using a Blue View Transilluminator (Vernier Biotechnology, Beaverton, OR, USA), excised, and purified using the QIAquickGel Extraction Kit (Qiagen, Valencia, CA, USA). The purified PCR products were TA-cloned into pCR2.1-TOPO using the TOPO TA Cloning kit (Thermo Fisher). Clones were transformed into chemically competent TOPO *Escherichia coli* and incubated for 1 h in SOC medium (Thermo Fisher) at 37 °C with agitation at 250 RPM. Aliquots of each culture were plated onto Luria-Bertani (LB) agar plates containing 0.1 mg/mL ampicillin and incubated at 37°C overnight. Colony PCR using vector-derived M13F and M13R primers was performed to identify colonies that harbored plasmids containing inserts of the expected size. Plasmids isolated from PCR-positive colonies using the PerfectPrep Spin Mini Kit (5 Prime, Gaithersburg, MD, USA) were sequenced using M13F and M13R primers by Genewiz (South Plainfield, NJ, USA) and the provided sequences were analyzed using the Lasergene 7.1 software package (DNASTAR, Madison, WI, USA).

### 2.3. In Silico Analyses and GenBank Accession Numbers of O. tsutsugamushi Strain ompA Sequences

*O. tsutsugamushi* strain *ompA* sequences were aligned using MegAlign and translated using EditSeq, both of which are part of the Lasergene 7.1 software package (DNASTAR). New *O. tsutsugamushi* strain *ompA* sequences identified herein have been deposited in GenBank with accession numbers listed in Table 1. Sequence distances and percent similarity of *O. tsutsugamushi* OmpA proteins were calculated using the ClustalW option in MegAlign. Heat maps indicating similarity/diversity of the *O. tsutsugamushi* strain OmpA nucleotide and translated protein sequences were generated using HEATMAP hierarchical clustering web tool (www.hiv.lanl.gov/content/sequence/HEATMAP/heatmap.html/). To predict a putative tertiary structure for *O. tsutsugamushi* Ikeda OmpA, the mature (minus the signal sequence) Ikeda sequence (residues 21 to 204) was threaded onto solved crystal structures of proteins with similar sequences using the PHYRE^2^ (Protein Homology/analogy Recognition Engine, version 2.0) server (www.sbg.bio.ic.ac.uk/phyre2) [66]. Six templates (c4zhvB (bacterial signaling protein Yfib), c5jirB (oop family OmpA-OmpF porin; *Treponema pallidum* protein tp0624), c3s0yA (periplasmic domain of motility protein B (MotB) residues 64-256), c2l26A (uncharacterized *Mycobacterium tuberculosis* membrane protein rv0899/mt0922), c2k1sA (folded C-terminal fragment of YiaD from *E. coli*), and c5wtlB (periplasmic portion of outer membrane protein 2a (OmpA) from *Capnocytophaga gingivalis*)) were selected to model OmpA based on heuristics to maximize confidence, percentage amino acid identity, and alignment coverage. For the final model, 92% of the protein was modeled at greater than 90% confidence. Residues that vary among the OmpA translated sequences identified in this study were denoted on the *O. tsutsugamushi* Ikeda OmpA PHYRE^2^ model using the PyMOL algorithm (pymol.org/educational).

### 2.4. Confirmation of ompA PCR Primer Specificity

Based on an alignment of all *ompA* sequences determined herein, primers *ompA*-57F and *ompA*-260R (Table 2) were designed to amplify nucleotides 57 to 260 based on *O. tsutsugamushi* Ikeda *ompA* (OTT_RS06375) [65]. To confirm that the primers were specific for *O. tsutsugamushi ompA*, they were utilized in PCR reactions that included genomic DNA from *Rickettsia* species (*R. africae*, *R. akari*, *R. australis*, *R. conorii*, *R. montanensis*, *R. parkeri*, *R. rhipicephali*, *R. rickettsii*, *R. sibirica, R. prowazekii*, *R. typhi*, and *R. amblyommii*), as well as other bacterial species (*Proteus mirabilis*, *E. coli*, *Legionella pneumophila*, *Bartonella vinsonii*, *Neorickettsia risticii*, *N. sennetsu, Francisella persica*, *A. phagocytophilum, E. chaffeensis*, *Coxiella burnetii*, *Chlamydia trachomatis*, and *Chlamydia pneumoniae*) using thermal cycling conditions and agarose gel electrophoresis described above. To verify that the control templates and thermal cycling conditions were amenable for PCR amplification, reactions were simultaneously performed on the *Rickettsia* species using degenerate primers that targeted the *Rickettsia* 17-kDa gene, R17K-135F and R17K-249R [36], and on the other bacterial species using primers that targeted a conserved eubacterial 16S rRNA sequence (Table 2) [36].

### 2.5. Mice

Female six- to eight-week old CD-1 Swiss outbred mice (Charles River Laboratories, Wilmington, MA, USA) were housed in animal biosafety level (ABSL)-2 laboratories prior to inoculation. Two days prior to inoculation, they were relocated to an ABSL-3 laboratory to adapt to their new surroundings. The mice were intradermally inoculated with 10^3^ MuID_50_ of *O. tsutsugamushi* Karp or Gilliam strains produced from liver-spleen homogenate of infected CD-1 mice into the dorsum of the right ear as previously described [67]. Sterile PBS was used as mock inoculum to inject negative control animals [67]. In some cases, Swiss CD-1 mice were intraperitoneally inoculated with 10^3^ MuID_50_ of *O. tsutsugamushi* Karp. At various days post-infection, the mice were euthanized, and organs or blood were harvested for DNA isolation [68]. All animal research was performed under the approval of the Institutional Animal Care and Use Committee at the Naval Medical Research Center (Protocol Number: 11-IDD-26).

### 2.6. Quantitative Real-Time PCR (qPCR)

To generate an *ompA* DNA standard, *ompA* nucleotides 1 to 615 were amplified using the OTT_RS06375-1F/615R primer set and Platinum HiFi Taq polymerase. The amplicon was gel-purified and cloned into pCR2.1-TOPO as described above. Concentration (in ng/µL) of the resulting plasmid, pCR2.1-*ompA*, was determined by UV spectrophotometry. The concentration was converted to copies/µL using the EndMemo DNA/RNA Copy Number Calculator (http://endmemo.com/bio/dnacopynum.php). To evaluate the sensitivity of the *ompA*-57F/260R primer set, triplicate 20 µL reactions containing either dilutions of pCR2.1-*ompA* ranging from 1 × 10^6^ to 1 × 10^−2^ copies/µL or no template were subjected to qPCR using SsoFastEvaGreenSupermix (Bio-Rad, Hercules, CA, USA) in a CFX96 Real-Time System thermocycler (Bio-Rad). Thermal cycling conditions consisted of an initial denaturation step of 95 °C for 3 min, followed by 40 cycles of 95 °C for 10 s and 55 °C for 30 s. The ability of the *ompA*-57F/260R primer set to detect *ompA* in DNA isolated from mouse tissues recovered from *O. tsutsugamushi* infected mice was also assessed. Using the DNeasy Blood and Tissue kit (Qiagen), DNA was isolated from the heart, kidney, liver, lung, spleen, and blood harvested on various days post-infection from Swiss CD-1 mice that had been infected with either *O. tsutsugamushi* Gilliam or Karp or uninfected control mice [68]. Fifty ng of mouse tissue DNA template per sample was subjected to qPCR exactly as described for the *ompA* DNA standards. Infrequently, an individual *ompA*-57F/260R reaction using uninfected mouse tissue DNA as template or no-template control would generate a Cq value at cycle 36.5 or later. In such experiments, only earlier Cq values generated for infected samples were considered as specifically amplifying *ompA*.

## 3. Results

### 3.1. Analyses of O. tsutsugamushi ompA DNA and Translated Amino Acid Sequences

**Figure 1:**
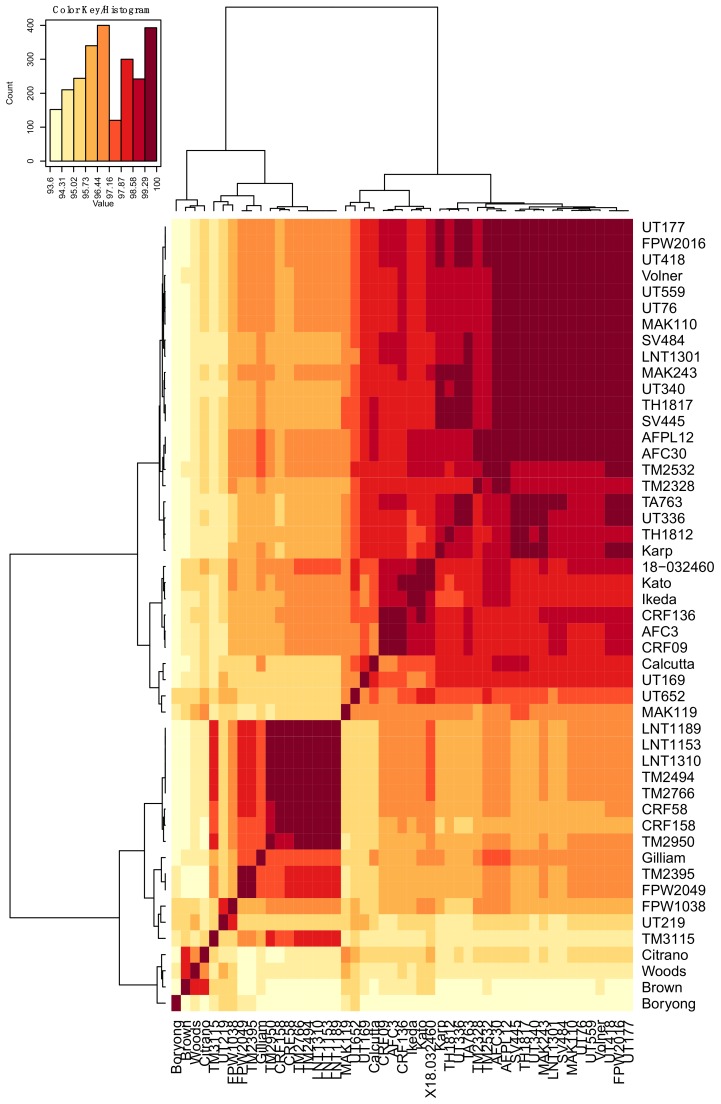
Heat map of percent nucleotide identity of the *ompA* DNA sequences among 49 *O. tsutsugamushi* isolates. The heat maps were generated using the pairwise identity matrix tables with hierarchical clustering method. The names of the isolates are provided on the right side and bottom of the heat map. Dendrograms are on the left side and on top of the heat map.

**Figure 2:**
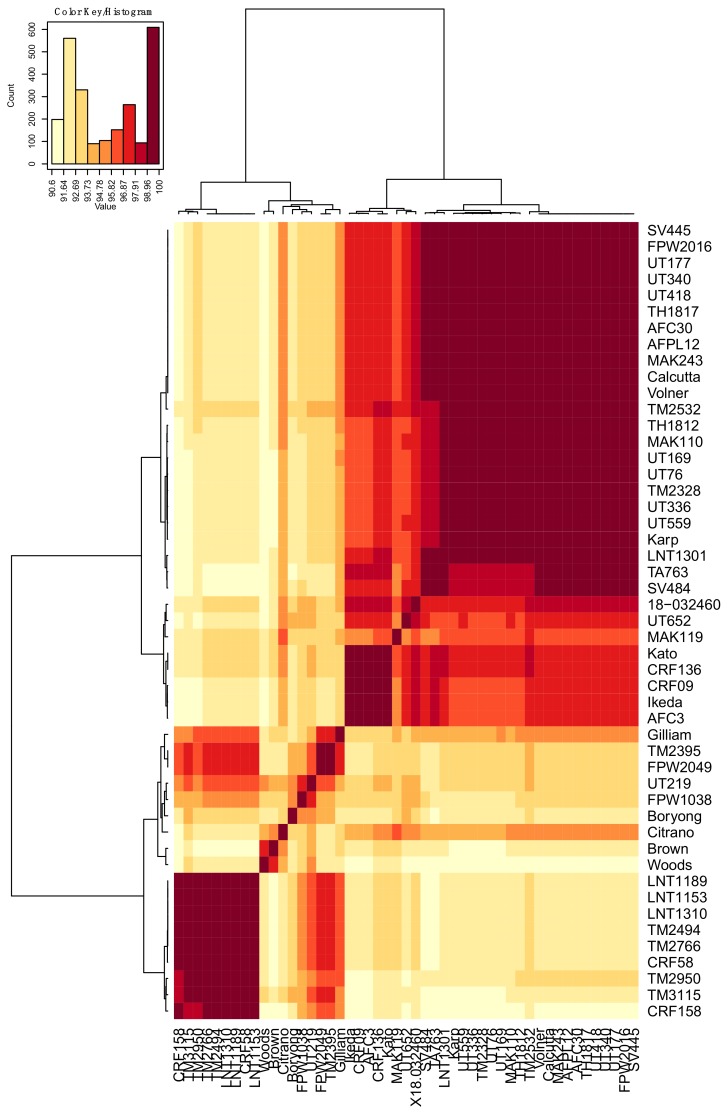
Heat map of percent identity of the OmpA translated amino acid sequences among 49 *O. tsutsugamushi* isolates. The heat maps were generated using the pairwise identity matrix tables with hierarchical clustering method. The names of the isolates are provided on the right side and bottom of the heat map. Dendrograms are on the left side and on top of the heat map.

**Figure 3:**
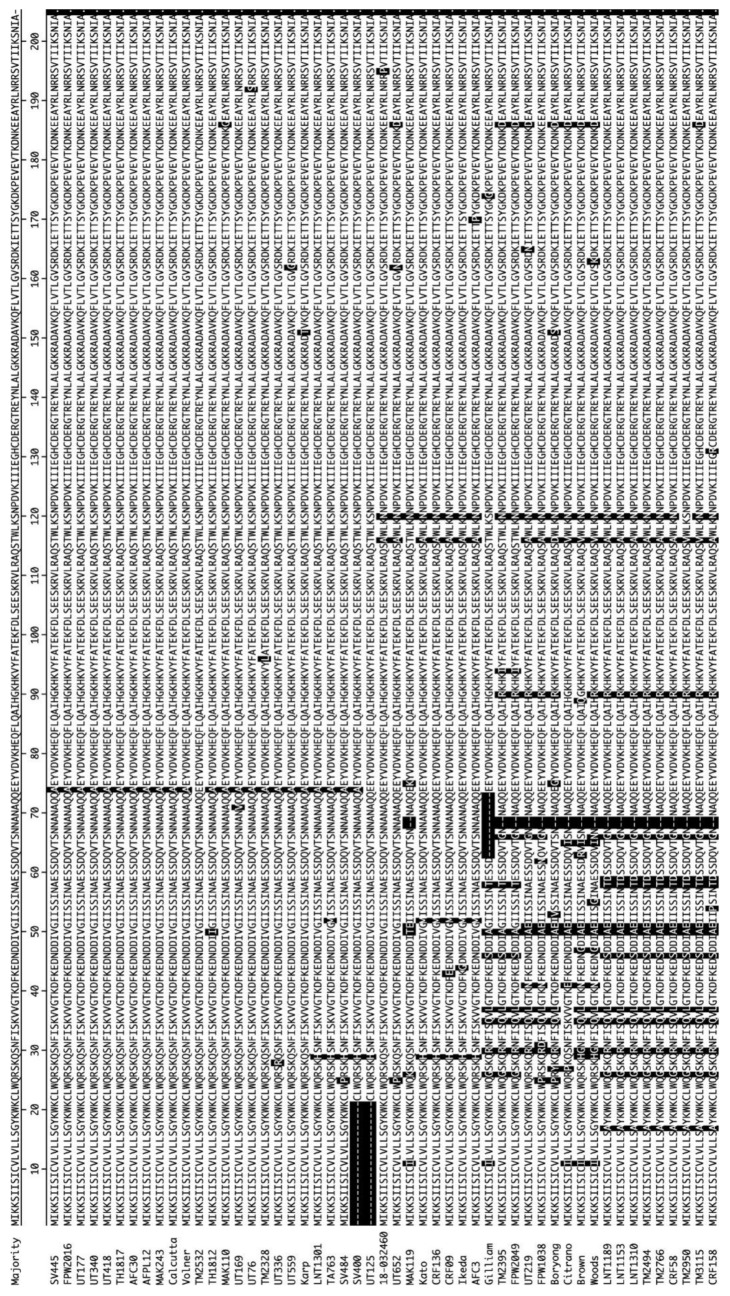
Alignment of translated *O. tsutsugamushi* isolate OmpA sequences. Presented is a ClustalW alignment of the translated amino acid sequences of all 51 *O. tsutsugamushi* isolates in this study. Amino acid differences relative to the majority (consensus) sequence are denoted by black-shaded white text. Sequence gaps relative to the majority are indicated by dashes. Residues 1–21 for SV400 and UT125 could not be predicted because *ompA* could be amplified by OTT_RS06375-64F/615R, but not OTT_RS06375-1F/615R.

DNA samples recovered from 51 geographically-diverse *O. tsutsugamushi* isolates (Table 1) were subjected to PCR using primer set OTT_RS06375-1F/615R (Table 2), which targets the full-length *ompA* gene (OTT_RS06375) of annotated *O. tsutsugamushi* strain Ikeda. [65]. Amplicons of the expected size were generated for all *O. tsutsugamushi* isolates except for SV400 and UT125. A second primer set specific for OTT_RS06375 nucleotides 64 to 615 successfully amplified the targeted *ompA* region from SV400 and UT125. Amplicons generated using the OTT_RS06375-1F/615R and OTT_RS06375-64F/615R primer sets were cloned and sequenced. The *ompA* nucleotide and translated amino acid sequences were deposited in GenBank. The nucleotide and amino acid identities ranged from 93.6% to 100.0% and 90.6% to 100.0%, respectively (Table S1, Figure 1 and Figure 2). SV400 and UT125 were excluded from heat map analyses because only partial sequences had been obtained for them. Aligning all OmpA amino acid sequences revealed that several segments thereof were 100% conserved among the isolates (Figure 3).

### 3.2. Molecular Modeling of OmpA

**Figure 4:**
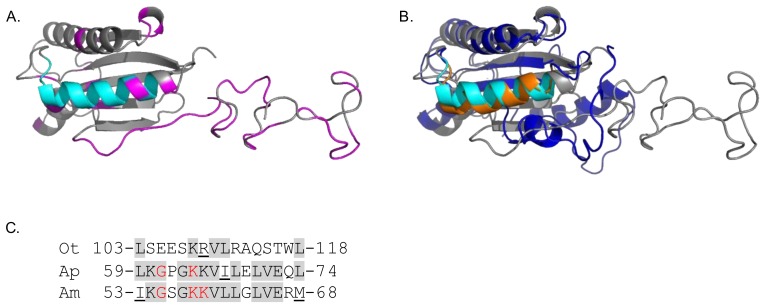
Molecular modeling of *O. tsutsugamushi* OmpA reveals structural similarity with *A. phagocytophilum* OmpA and conservation of residues that are critical for *Anaplasma* spp. OmpA function. (**A**) Predicted tertiary structure for *O. tsutsugamushi* OmpA. The OmpA mature protein sequence was predicted using the PHYRE^2^ algorithm. The cyan portion corresponds to residues 103 to 118, which are analogous to residues 59 to 74 and 53 to 68 of *A. phagocytophilum* and *A. marginale* OmpA predicted tertiary structures, respectively, that form receptor-binding domains. Magenta residues are those that vary among the 51 *O. tsutsugamushi* OmpA sequences studied herein. (**B**) Overlay of *O. tsutsugamushi* and *A. phagocytophilum* predicted OmpA tertiary structures. *A. phagocytophilum* OmpA residues are labeled dark blue except for residues 59 to 74 (labeled orange) that lie within a surface-exposed alpha helix and constitute the receptor binding domain. *O. tsutsugamushi* OmpA residues are labeled gray except for residues 103 to 118 (labeled cyan) that are analogous to *A. phagocytophilum* OmpA residues 59 to 74. (**C**) Alignment of *O. tsutsugamushi* (Ot) OmpA residues 103 to 118, *A. phagocytophilum* (Ap) amino acids 59 to 74, and *A. marginale* (Am) residues 53 to 68. Identical residues among the three sequences are shaded gray. Similar amino acids are underlined. Functionally essential residues in the *Anaplasma* spp. OmpA proteins are in red text.

OmpA proteins of *A. phagocytophilum* and *A. marginale*, which are in the order Rickettsiales with *O. tsutsugamushi*, contribute to their abilities to bind and invade mammalian host cells [41,44]. We previously demonstrated that these two OmpA proteins’ tertiary structures are highly similar and residues that are critical for adhesin function are conserved between them and are presented as part of surface-exposed alpha helices [41,44,45]. To predict the tertiary structure of *O. tsutsugamushi* OmpA, molecular modeling of Ikeda OmpA residues 21 to 204 (minus the signal sequence) as a representative naturally-occurring OmpA sequence was performed using the PHYRE^2^ recognition server (www.sbg.bio.ic.ac.uk/phyre2/html/page.cgi) [69], which generates three-dimensional models for protein sequences and threads them on known crystal structures. The resulting model is presented in Figure 4A. The *O. tsutsugamushi* OmpA tertiary structure was evaluated for similarity to *Anaplasma* spp. OmpA. Because the *A. phagocytophilum* and *A. marginale* OmpA models are nearly identical [41], the *O. tsutsugamushi* OmpA predicted structure was compared only to that of *A. phagocytophilum*. Threading the two models onto each other using PyMOL revealed their folded portions to be very similar structurally with the exception of *O. tsutsugamushi* residues 21 through 50, which are disordered (Figure 4B). Notably, *O. tsutsugamushi* OmpA bears a surface-exposed alpha helix that overlays with the functionally-essential surface-exposed alpha helix of *A. phagocytophilum* OmpA. Moreover, aligning the *A. phagocytophilum* (L_59_KGPGKKVILELVEQL_74_) and *A. marginale* OmpA binding domains (I_53_KGSGKKVLLGLVERM_68_) with the analogous region of *O. tsutsugamushi* OmpA (L_103_SEESKRVLRAQSAWL_118_) indicated conservation of several residues, including a lysine that is critical for *A. phagocytophilum* OmpA and *A. marginale* OmpA adhesin function [41,45] (Figure 4C). This region is identical among all *O. tsutsugamushi* translated OmpA sequences in this study with the exception of residue 116 (Figure 3 and Figure 4). Thus, *O. tsutsugamushi* OmpA is predicted to exhibit high structural similarity to OmpA proteins of other Rickettsiales members that are important for infection, its surface-exposed alpha helix is identical among all *O. tsutsugamushi* isolates examined herein, and residues of the alpha helix are identical to those of *Anaplasma* spp. OmpA proteins that are key for pathogenicity.

### 3.3. Development of a Primer Set That Specifically Amplifies an ompA Sequence Unique to O. tsutsugamushi

**Figure 5:**
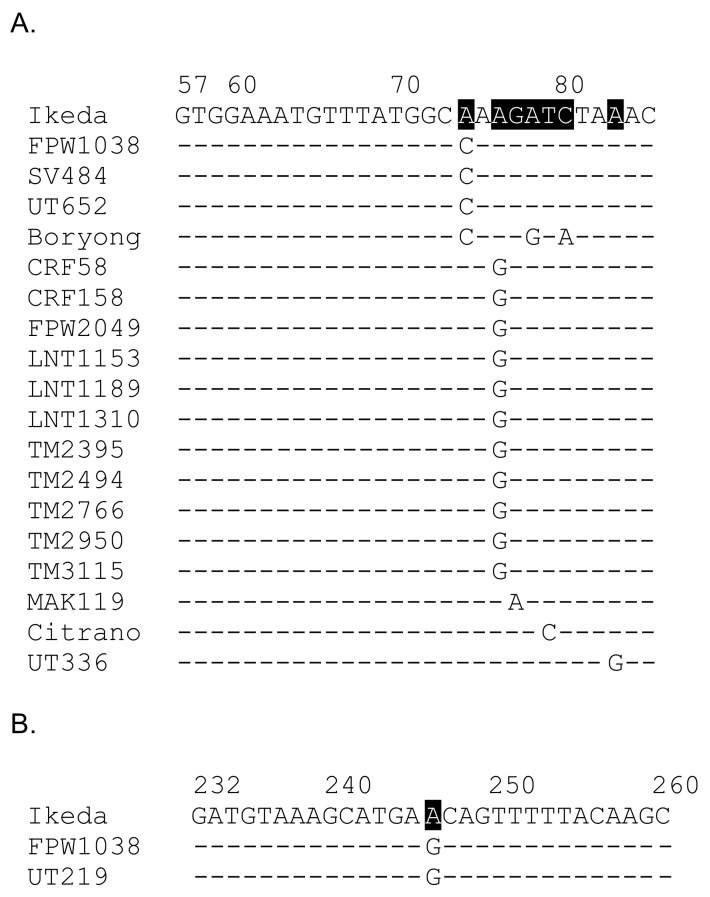
Nucleotide mismatches in the binding sites for *ompA*-57F and *ompA*-260R among *O. tsutsugamushi* isolates examined in this study. The *ompA*-57F (**A**) and *ompA*-260R (**B**) sequences, both of which correspond to *O. tsutsugamushi* Ikeda nucleotides, are listed. Below each are the corresponding nucleotides of where each primer would bind for any isolate in this study that has at least one mismatch. Numbers above the sequences refer to the nucleotide positions in Ikeda *ompA*. Dashes indicate identical nucleotides to those of *ompA*-57F and *ompA*-260R. Mismatches in the primer sequences are denoted by black-shaded white text with the specific nucleotide difference indicated per sequence below.

Next, whether a primer set could be devised that would amplify a segment of *O. tsutsugamushi ompA* from all *O. tsutsugamushi* isolates in this study was examined. A BLASTN search against GenBank using the *O. tsutsugamushi* Ikeda OTT_RS06375 sequence as query determined that nucleotides 64 to 279 were unique to *O. tsutsugamushi* isolates. Examination of nucleotides flanking and within this region for sequences that would have annealing temperatures compatible with thermal cycling conditions denoted nucleotides 57 to 85 (primer *ompA*-57F; Table 2) and 232 to 260 (primer *ompA*-260R; Table 2) as potential primers. For nucleotides 57 to 85, 32 of the 51 isolates had a sequence that was identical to the consensus, 17 had a single nucleotide mismatch, and one (Boryong) had three nucleotide mismatches (Figure 5A). For isolates SV400 and UT125, which *ompA* sequences were identified using primer set OTT_RS06375-64F/615R and therefore began with nucleotide 64, identity of nucleotides 57 to 63 could not be determined. However, nucleotides 64 to 85 for these two isolates exactly matched the consensus. All isolates exhibited 100% identity among nucleotides 232 to 260 except for two (FPW1038 and UT219), each of which had a single nucleotide mismatch (Figure 5B). For all isolate target sequences of the *ompA*-57F/260R primer set that had nucleotide mismatches, only one (UT336) exhibited a nucleotide mismatch near the 3′ end of either primer. Importantly, the UT336 nucleotide mismatch for *ompA*-57F did not occur within the final two nucleotides. Thus, it was expected that the primer set would amplify *ompA* from all 51 isolates.

**Figure 6:**
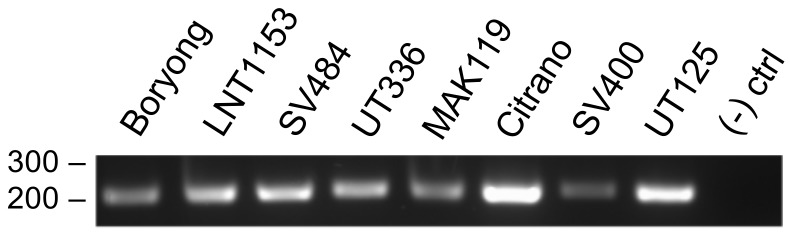
Primer set *ompA*-57F/260R amplifies *ompA* sequences having one to three nucleotide mismatches in the primer binding sites. DNA isolated from tissue culture cells infected with each of the indicated *O. tsutsugamushi* isolates or minus template control ((−) ctrl) were subjected to PCR analysis using *ompA*-57F/260R primers. The numbers to the left of the panel correspond to DNA ladder sizes. Data are representative of three experiments with similar results.

**Figure 7:**
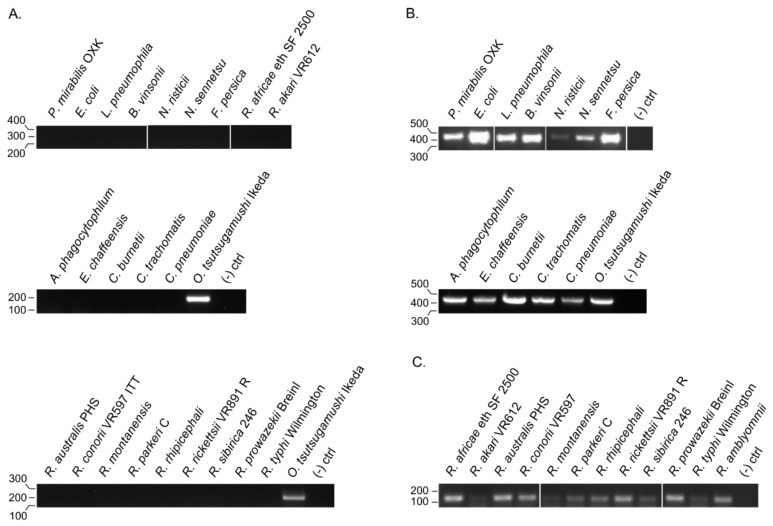
Confirmation of *ompA*-57F/260R specificity. DNA samples from eubacterial and *Rickettsia* spp. were subjected to PCR using (**A**) *ompA*-57F/260R. The eubacterial and *Rickettsia* spp. DNA samples were also subjected to PCR using primer sets targeting eubacterial 16S rRNA (**B**) and the rickettsial gene encoding the 17-kDa protein (**C**) to verify sample integrity. For the experiment in (A), *O. tsutsugamushi* Ikeda DNA was included as a positive control. A minus template control ((−) ctrl) was included in each experiment. Vertical lines between lanes indicate that irrelevant lanes from the gel images were removed. Data are representative of two experiments with similar results.

To confirm the efficacy of the *ompA*-57F/260R primer set, it was evaluated for the ability to amplify its target from six representative isolates that had one or more nucleotide mismatches at the primer binding sites (Boryong, LNT1153, SV484, UT336, MAK119, Citrano) plus SV400 and UT125, for which it was unknown whether *ompA*-57F would bind. A band of the expected size was generated for all eight samples, but not for negative control reaction that lacked DNA template (Figure 6). Next, whether the primer set would non-specifically generate products from a variety of eubacterial human pathogens, including *A. phagocytophilum*, *C. burnetii*, *C. trachomatis*, *C. pneumoniae*, and several *Rickettsia* spp. was examined. The primer set failed to yield PCR products from the 23 samples examined but produced an amplicon of the expected size from *O. tsutsugamushi* Ikeda (Figure 7A), which has a sequence that is identical to the consensus binding sites for both primers. Because PCR products could be generated from the eubacterial spp. using primers targeting eubacterial 16S rRNA and the rickettsial gene encoding 17-kDa protein [36] (Figure 7B,C), it could be concluded that the integrity of these samples was sufficient to allow for DNA amplification and that the only reason the *ompA*-57F/260R primer set did not generate PCR products for them was specifically due to disparity in the primer binding sites. Overall, these data demonstrate that the *ompA*-57F/260R specifically amplifies its DNA target sequence and does so even if it contains limited nucleotide mismatches.

### 3.4. Evaluation of the O. tsutsugamushi ompA-Specific Primer Set in qPCR

**Figure 8:**
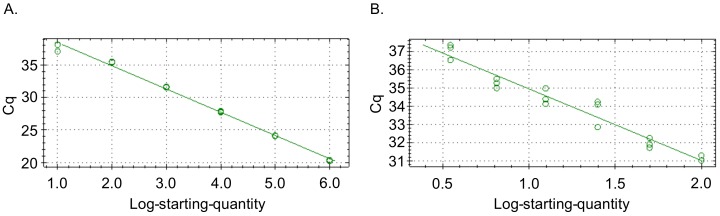
Primer set specific for *O. tsutsugamushi* OmpA is capable of amplifying low copy number *ompA* DNA standards. Standard curves generated by *ompA*-57F/260R amplification of *ompA* DNA standards diluted ten-fold from 1 × 10^6^ to 1 × 10^−2^ (**A**) and from 100 to 3.5 copies (**B**). Data are representative of two experiments with similar results.

To determine the detection limit of *ompA*-57F/260R, the primer set was evaluated by qPCR using plasmid pCR2.1-*ompA* as template, which has *ompA* nucleotides 1 to 615 inserted, serially-diluted from 1 × 10^6^ to 1 × 10^−2^ copies per reaction. The primers detected *ompA* as low as 10^1^ copies (R^2^ = 0.995) (Figure 8A). The experiment was repeated with reactions containing pCR2.1-*ompA* diluted from 100 to 3.5 copies. The primers detected *ompA* DNA at a concentration as low as 3.5 copies (R^2^ = 0.950) (Figure 8B). Thus, *ompA*-57F/260R has an approximate detection limit of 3.5 copies.

**Figure 9:**
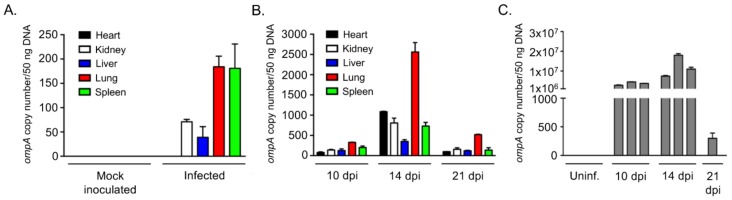
The *ompA*-57F/260R primer set amplifies *ompA* in qPCR from DNA recovered from the organs or blood of *O. tsutsugamushi*-infected mice. Fifty ng of DNA isolated on the indicated day post-infection (dpi) from the noted organs (**A**,**B**) or blood (**C**) from Swiss CD-1 mice that had been mock inoculated or that had been infected with *O. tsutsugamushi* Gilliam (**A**) or Karp (**B**,**C**). Each bar represents the mean + SD *ompA* copy number per 50 ng DNA recovered from an individual mouse analyzed in triplicate. Data are representative of at least three experiments with similar results.

Next, the *ompA*-57F/260R primer set was evaluated using qPCR for the ability to detect and quantify *ompA* copies in DNA samples isolated from organs harvested from Swiss CD-1 mice infected with *O. tsutsugamushi* Gilliam. The CD-1 mouse intradermal inoculation model was recently demonstrated to exhibit features of early scrub typhus infection in humans, including distant organ dissemination. Gilliam was one such strain that was evaluated using this model [68]. Reactions performed on the same plate with pCR2.1-*ompA* diluted ten-fold from 1 × 10^6^ to 1 × 10^0^ copies allowed for copy number quantitation. Negative control reactions consisted of those containing DNA isolated from organs of mock-inoculated mice and those lacking DNA template. The primers detected *ompA* at copy numbers of 71.0 ± 5.2, 39.3 ± 22.2, 184.0 ± 22.2, and 181.0 ± 49.8 in kidney, liver, lung, and spleen DNA samples, respectively, recovered on Day 6 from a mouse that had been intradermally injected with *O. tsutsugamushi* Gilliam, but failed to amplify *ompA* from DNA isolated from heart of the infected mouse or from a mock-inoculated mouse (Figure 9A). When qPCR was performed on DNA samples isolated on Days 10, 14, and 21 from organs of mice that had been intradermally inoculated with *O. tsutsugamushi* Karp, *ompA* DNA was detected in all samples and at the highest levels for each organ in samples isolated on day 14 (Figure 9B). Copy numbers of *ompA* for these samples ranged from 22.4 ± 5.0 (heart 10 days post-infection) to 1890 ± 219.0 (lung 14 days post-infection). Lungs having the highest level of *ompA* DNA is consistent with the bacterial burden being the greatest in the lungs in this mouse model [68]. The primer sets also detected *ompA* DNA in blood drawn from mice that had been intraperitoneally inoculated with the Karp strain, again with Day 14 samples having the highest *ompA* levels (Figure 9C). Intraperitoneal inoculation resulted in a high bacteremia. Only one of three mice survived until Day 21 following the intraperitoneal inoculation route. These data demonstrate the ability of *ompA*-57F/260R to amplify and quantify *ompA* copies in DNA samples recovered from organs and blood of *O. tsutsugamushi*-infected mice.

## 4. Discussion

Scrub typhus is a global health concern for which neither a vaccine that provides heterologous protection nor a reliable diagnostic assay exists. To effectively protect against or detect the diversity of *O. tsutsugamushi* strains, the bacterial target must be highly conserved. OmpA satisfies this criterion, as it displays 93.6% to 100.0% and 90.6% to 100.0% conservation at the nucleotide and protein levels, respectively, among the isolates in this study that originated from multiple Asia-Pacific locations.

While the role of OmpA in *O. tsutsugamushi* pathobiology is unclear, studies of other Rickettsiales members’ OmpA proteins offer precedents that *O. tsutsugamushi* OmpA likely contributes to and could be immunologically targeted to inhibit infection. OmpA proteins of *A. phagocytophilum*, *A. marginale*, *E. chaffeensis*, and *R. conorii* are each on the bacterial surface, participate in host cell entry, and can be targeted by antibodies to inhibit infection in vitro [38,41,42,44,45]. Patients naturally infected with *A. phagocytophilum* or *R. conorii* and animals experimentally infected with *A. phagocytophilum* or *E. chaffeensis* develop antibodies that recognize recombinant forms of the respective OmpA proteins in serological assays [38,44,46], indicating that Rickettsiales bacteria express OmpA during in vivo infection. The abilities of *A. phagocytophilum* and *A. marginale* OmpA to mediate bacterial adhesion to, and invasion of, host cells, rely on receptor-binding domains that consist of specific lysine and glycine residues within the proteins’ structurally-conserved, surface-exposed alpha helices. Antisera specific for these binding domains inhibits *Anaplasma* spp. infection of host cells [41,45]. Strikingly, the predicted *O. tsutsugamushi* OmpA tertiary structure is very similar to that of *A. phagocytophilum* OmpA, so much so that their aforementioned alpha-helices and a lysine residue thereof overlay when the proteins are superimposed on each other. It will be important to confirm whether *O. tsutsugamushi* OmpA is surface-exposed and if antiserum raised against the full-length protein or its alpha helix amino acids 103–118 inhibits infection of host cells.

A *R. prowazekii ompA* deletion mutant retains the ability to productively infect mice [70], likely due to OmpA being one of many OMPs that cooperatively function to mediate invasion of host cells [71,72,73]. Nonetheless, it is worth investigating whether immunization against OmpA offers protection from *O. tsutsugamushi* challenge. A humoral immune response against OmpA or a key portion thereof together with other conserved OMPs could inhibit bacterial entry into host cells, which would essentially lead to its demise due to its obligatory intracellular lifestyle. This very concept has been demonstrated for blocking *A. phagocytophilum* infection in vitro: whereas OmpA binding domain antibody alone reduces infection by approximately 25%, an antibody cocktail targeting the binding domains of OmpA together with two additional OMPs nearly eliminates infection of host cells [44,45]. Anti-OmpA antibodies could also eradicate *O. tsutsugamushi* load in vivo via complement-mediated killing or opsonophagocytosis. Indeed, although the exact mechanism is unclear, guinea pigs immunized with a recombinant form of truncated *R. heilongjiangensis* OmpA exhibited reduced bacterial load, organ pathology, and interstitial pneumonia following challenge with *R. heilongjiangensis* or *R. rickettsii*, compared to sham-immunized animals [49].

Exploiting the high degree of *ompA* nucleotide conservation facilitated development of primer set *ompA*-57F/260R, which specifically amplified its target from all *O. tsutsugamushi* isolates examined herein, including *ompA* sequences having up to three nucleotide mismatches in the primer binding sites. In qPCR, *ompA*-57F/260R detected *ompA* at a copy number as low as 3.5. This sensitivity level rivaled or exceeded that reported for other qPCR assays [34,74,75]. Moreover, these primers detected *ompA* in DNA isolated from organs or blood of *O. tsutsugamushi*-infected mice. The ability of *ompA*-57F/260R to detect *ompA* in the presence of excess host tissue-derived DNA evidences its potential for sensitively detecting *O. tsutsugamushi ompA* in DNA isolated from scrub typhus patient-derived samples such as eschar swabs, blood, or buffy coats, as has been demonstrated for other qPCR assays [34,74,76,77,78,79,80,81].

## 5. Conclusions

The high degree of nucleotide and amino acid conservation of OmpA among diverse *O. tsutsugamushi* isolates and its structural similarity to other Rickettsiales OmpA proteins that have been successfully targeted to inhibit bacterial invasion of host cells, argue for its consideration as a vaccine candidate that could provide heterologous protection and as a molecular target that could be useful in diagnosing scrub typhus infections.

## Figures and Tables

**Table 1:** *O. tsutsugamushi* isolates used in this study.

Isolate	Geographic Origin	Reference	*ompA* GenBank Accession Number or Locus Tag
18-032460	Malaysia	[63]	MH167971
AFC3	Thailand	[56]	MH167972
AFC30	Thailand	NR	MH167973
AFPL12	Thailand	NR	MH167974
Boryong	South Korea	[54]	OTBS_RS08365
Brown	Australia	[61]	MH167975
Calcutta	India	NR	MH167976
Citrano	Australia	[61]	MH167977
CRF09	Northern Thailand	[52]	MH167978
CRF58	Northern Thailand	[52]	MH167979
CRF136	Northern Thailand	[52]	MH167980
CRF158	Northern Thailand	[52]	MH167981
FPW1038	Western Thailand	[53]	MH167982
FPW2016	Western Thailand	[53]	MH167983
FPW2049	Western Thailand	[53]	MH167984
Gilliam	Burma	[57]	MH167985
Ikeda	Japan	[58]	OTT_RS06375
Karp	New Guinea	[55]	OTSKARP_0358
Kato	Japan	[64]	OTSKATO_0610
LNT1153	Northwestern Laos	[51]	MH167986
LNT1189	Northwestern Laos	[51]	MH167987
LNT1301	Northwestern Laos	[51]	MH167988
LNT1310	Northwestern Laos	[51]	MH167989
MAK110	Taiwan	[62]	MH167990
MAK119	Taiwan	[62]	MH167991
MAK243	Taiwan	[62]	MH167992
SV400	Southern Laos	[51]	MH167993
SV445	Southern Laos	[51]	MH167994
SV484	Southern Laos	[51]	MH167995
TA763	Thailand	[59]	OTSTA763_0977
TH1812	Thailand	[59]	MH167996
TH1817	Thailand	[59]	MH167997
TM2328	Central Laos	[51]	MH167998
TM2395	Central Laos	[51]	MH167999
TM2494	Central Laos	[51]	MH168000
TM2532	Central Laos	[51]	MH168001
TM2766	Central Laos	[51]	MH168002
TM2950	Central Laos	[51]	MH168003
TM3115	Central Laos	[51]	MH168004
UT76	Northeastern Thailand	[53]	MH168005
UT125	Northeastern Thailand	[53]	MH168006
UT169	Northeastern Thailand	[53]	MH168007
UT177	Northeastern Thailand	[53]	MH168008
UT219	Northeastern Thailand	[53]	MH168009
UT336	Northeastern Thailand	[53]	MH168010
UT340	Northeastern Thailand	[53]	MH168011
UT418	Northeastern Thailand	[53]	MH168012
UT559	Northeastern Thailand	[53]	MH168013
UT652	Northeastern Thailand	[53]	MH168014
Volner	New Guinea	[60]	MH168015
Wood	Australia	[61]	MH168016

NR: no previous published report.

**Table 2:** Oligonucleotide primers used in this study.

Primer Designation ^a^	Sequence (5′ to 3′)
OTT_RS06375-1F	ATGATTAAAAAGTCAATTATTAGTATATGTGTATTAGTGC
OTT_RS06375-615R	CTATGCTATATTACTTTTAATAATTGTGACAGACC
OTT_RS06375-64F	TGTTTATGGCAAAGATCTAAACATAGTAAC
*ompA*-57F	GTGGAAATGTTTATGGCAAAGATCTAAAC
*ompA*-260R	GCTTGTAAAAACTGTTCATGCTTTACATC
Eubacterial 16S-F	GTTCGGAATTACTGGGCGTA
Eubacterial 16S-R	AATTAAACCGCATGCTCCAC
R17K-135	ATGAATAAACAACGK^b^CANGGHACAC
R17K-249	RAAGTAATGCRCCTACACCTACTC

^a^ F and R refer to primers that bind to the sense and antisense strand, respectively. The number immediately preceding the F or R denotes the first nucleotide position where the primer binds. ^b^ Degenerate positions contained equal molar base concentrations of the following nucleotides: (K), guanine and thymine, (N), adenine, guanine, thymine, and cytosine; (H), adenine, guanine, and thymine; (R) adenine and guanine.

## Supplementary Materials

The following are available online at http://www.mdpi.com/2414-6366/3/2/63/s1, Table S1: Nucleotide and amino acid identity values for *O. tsutsugamushi* OmpA sequences.
